# Persistent hyperinsulinemic hypoglycemic in infant: a case report

**DOI:** 10.1186/1687-9856-2015-S1-P121

**Published:** 2015-04-28

**Authors:** Nur Rochmah, Muhammad Faizi

**Affiliations:** 1 Faculty of Medicine-Airlangga University-dr Soetomo Hospital, Surabaya, East Java, Indonesia

## 

Persistent hyperinsulinemic hypoglycemic in infant is risk factor for impairment during brain development process. Early diagnosis and treatment will result in better outcome. The aim is to report a case of persistent hyperinsulinemic hypoglycaemic infant. Methode is case report. A, 2 days old term infant, was referred due to hypoglicemia. He got generalized seizure and recurrent hypoglicemia. He can drink well. No vomiting observed. The Apgar score was 6-7-8. Birth weight was 4000 gram. He was the third child. Second child had the same history. No history of diabetes mellitus in the family. Physical examination revealed normal limit. Laboratory examination showed Hb 16.8 gram/dl, WBC17.4/cmm; hematocrite 50.1%; platelet 316/cmm; blood glucose 105 gram/dl, potassium 4.6 mmol/l, sodium 139 mmol/l, chloride 106 mmol/l, calsium 9.6 mg/dl, BUN 5.1 mg/dl, creatinin serum 0.82 mg/dl, Direct Bilirubin 0.079 mg/dl, Total bilirubin 0.19 mg/dl, SGOT 16 U/L, SGPT 10 U/L, CRP 11.10 mg/dl. During hypoglycaemia we got the result as follows: growth hormon 2.99 ng/ml (0.06-5), cortisol serum 198 ng/ml (50-250), fasting Insulin 10.30 Uu/ml (2.6-24.9). Head ultrasonography revealed normal. The patient was given IVFD Dextrose 10 0.18% saline (glucose infusion rate 4-5 mg/kg/min), breast milk 8x30-60cc, Ocreotide 5 mcg/kg/day iv, Nifedipin 4x 0.5-2.5mg per oral. Bolus 2cc/kg body weight of D10% if the blood sugar level was low. Hypoglycemic improved after treatment. As conclusion we should be aware of hypoglycemia in infant, it may due to persistent hyperinsulinism hypoglycemia of infant in which careful management is needed.

Written informed consent was obtained from each patient's parent or guardian for publication of this abstract and any accompanying images. A copy of the written consent is available for review by the Editor of this journal.

